# Bioaugmentation of *Lactobacillus delbrueckii* ssp. *bulgaricus* TISTR 895 to enhance bio-hydrogen production of *Rhodobacter sphaeroides* KKU-PS5

**DOI:** 10.1186/s13068-015-0375-z

**Published:** 2015-11-25

**Authors:** Sucheera Laocharoen, Alissara Reungsang, Pensri Plangklang

**Affiliations:** Department of Biotechnology, Faculty of Technology, Khon Kaen University, Khon Kaen, 40002 Thailand; Research Group for Development of Microbial Hydrogen Production Process from Biomass, Khon Kaen University, Khon Kaen, 40002 Thailand

**Keywords:** Bio-hydrogen, Purple non-sulfur photosynthetic bacteria, Dark fermentative bacteria, Lactic acid-producing bacteria

## Abstract

**Background:**

Bioaugmentation or an addition of the desired microorganisms or specialized microbial strains into the anaerobic digesters can enhance the performance of microbial community in the hydrogen production process. Most of the studies focused on a bioaugmentation of native microorganisms capable of producing hydrogen with the dark-fermentative hydrogen producers while information on bioaugmentation of purple non-sulfur photosynthetic bacteria (PNSB) with lactic acid-producing bacteria (LAB) is still limited. In our study, bioaugmentation of *Rhodobacter sphaeroides* KKU-PS5 with *Lactobacillus delbrueckii* ssp. *bulgaricus* TISTR 895 was conducted as a method to produce hydrogen. Unfortunately, even though well-characterized microorganisms were used in the fermentation system, a cultivation of two different organisms in the same bioreactor was still difficult because of the differences in their metabolic types, optimal conditions, and nutritional requirements. Therefore, evaluation of the physical and chemical factors affecting hydrogen production of PNSB augmented with LAB was conducted using a full factorial design followed by response surface methodology (RSM) with central composite design (CCD).

**Results:**

A suitable LAB/PNSB ratio and initial cell concentration were found to be 1/12 (w/w) and 0.15 g/L, respectively. The optimal initial pH, light intensity, and Mo concentration obtained from RSM with CCD were 7.92, 8.37 klux and 0.44 mg/L, respectively. Under these optimal conditions, a cumulative hydrogen production of 3396 ± 66 mL H_2_/L, a hydrogen production rate (HPR) of 9.1 ± 0.2 mL H_2_/L h, and a hydrogen yield (HY) of 9.65 ± 0.23 mol H_2_/mol glucose were obtained. KKU-PS5 augmented with TISTR 895 produced hydrogen from glucose at a relatively high HY, 9.65 ± 0.23 mol H_2_/mol glucose, i.e., 80 % of the theoretical yield.

**Conclusions:**

The ratio of the strains TISTR 895/KKU-PS5 and their initial cell concentrations affected the rate of lactic acid production and its consumption. A suitable LAB/PNSB ratio and initial cell concentration could balance the lactic acid production rate and its consumption in order to avoid lactic acid accumulation in the fermentation system. Through use of appropriate environmental conditions for bioaugmentation of PNSB with LAB, a hydrogen production could be enhanced.

**Electronic supplementary material:**

The online version of this article (doi:10.1186/s13068-015-0375-z) contains supplementary material, which is available to authorized users.

## Background

Hydrogen is a valuable alternative fuel and energy carrier as it has a high energy content, 122 kJ/g, which is 2.75 times higher than that of hydrocarbon fuels [1]. Biological hydrogen production is considered an environmentally friendly process because the operations to produce hydrogen from biomass and waste materials are conducted under mild conditions [2, 3]. In addition, only water is formed when hydrogen is burned as a fuel or is used to generate electricity [4]. Dark and photo-hydrogen production are the primary methods of biological hydrogen production. In dark fermentation, the fermentative bacteria used organic substances as the sole source of energy and electrons for the metabolic activities and hydrogen production, while photo-fermentation uses organic substrates as electron donors and light as an energy source [5, 6].

Photo-fermentative hydrogen production was found in purple non-sulfur photosynthetic bacteria (PNSB). The photosystem of PNSB is located in the cell membrane and contains light-harvesting pigments, bacteriochlorophyll and carotenoids, as well as an associated electron transport chain [7]. Light energy is absorbed by two light-harvesting complexes that are referred as “core” (light-harvesting complex 1; LH1) and “peripheral” (light-harvesting complex 2; LH2) antenna complexes. These two complexes are channeled into the reaction complex which initiates a cyclic electron flow through several electron carriers [6, 7]. The electrochemical proton gradient is generated during the photosynthetic process of PNSB. A proton gradient is used to drive adenosine triphosphate (ATP) synthesis by ATP synthase and is also used to produce a reduced ferredoxin [Fd_(red)_] by reversed electron flow [6, 8]. ATP and Fd_(red)_ are needed for reducing protons to hydrogen by the activity of nitrogenase [6, 7], which is the key enzyme for the hydrogen production process of PNSB. The nitrogenase side reaction is showed in Eq. (1). The electron sink pathways for competing the hydrogen production process by PNSB are carbon dioxide fixation and polyhydroxybutyrate (PHB) synthesis [5].

1 $$2H^{ + } + Fd_{{(red)}} (2e^{ - } ) + 4ATP\xrightarrow[{Nitrogenase}]{}H_{2} + 4\left( {ADP + P_{i} } \right)$$

The improvement of the efficiency of hydrogen production can be conducted using various approaches such as genetic engineering to introduce the hydrogen generation pathway and to eliminate the competition metabolisms with hydrogen production of microorganism [9, 10] as well as the integration of dark and photo-fermentation system [11–15]. In addition, the bioaugmentation or an addition of the desired microorganisms or specialized microbial strains into the anaerobic digesters can enhance the performance of microbial community in the hydrogen production process. Previous researches demonstrated the effectiveness of bioaugmentation in improving hydrogen production [16–23]. Kuo et al. [17] found that a hydrogen production potential in a 2-phase bio-hydrogen and bio-methane production from vegetable-based kitchen waste and Napier grass increased drastically after the bioaugmentation of *Clostridium* sp. TCW1. Marone et al. [18] reported that the bioaugmentation of indigenous microbial communities in vegetable waste with three hydrogen-producing strains viz., *Buttiauxella* sp. 4, *Rahnella s*p. 10, and *Raoultella* sp. 47 using each single strain and mixed three strains together significantly increased the hydrogen yield (HY) and the hydrogen production rate (HPR) in comparison to non-bioaugmentation. Bioaugmentation would not only improve the hydrogen production process but also can be used to overcome the inhibition occurred in the hydrogen production process. Goud et al. [16] reported that the bioaugmentation of native acidogenic microflora with *Bacillus subtilis*, *Pseudomonas stutzeri,* and *Lysinibacillus fusiformis* could increase substrate degradation rate and enhance fermentative hydrogen production from real-field food wastewater at elevated organic load. In addition, the bioaugmentation strategies could shorten the digestion time in a bioreactor. For example, Ma et al. [19] found that the bioaugmentation of the activated sludge with mixed cultures of specialized bacteria consisting of *Pseudomonas*, *Bacillus*, *Acinetobacter*, *Flavobacterium*, and *Micrococcus* in a contact oxidation process decreased the chemical oxygen demand (COD) and ammonia nitrogen (NH^4+^-N) from 320–530 mg/L and 8–25 mg/L to below 80 and 10 mg/L, respectively, within 20 days, while the un-bioaugmented conventional activated sludge process spent 30 days. From the discussion above, we can see that the bioaugmentation methods are successfully used in dark hydrogen fermentation process or methane production process, while there is very limited information on the application of bioaugmentation in the dark- and photo-hydrogen fermentation processes.

In dark- and photo-hydrogen fermentation processes, a dark fermentation is done before photo fermentation in which effluent of the dark fermentation is used as substrate by photo-fermentative bacteria in a second reactor. Dark fermentation by acidogenic–anaerobic bacteria produces hydrogen concomitantly with soluble metabolites, i.e., volatile fatty acids (VFAs) and alcohols [24]. The VFAs obtained from a dark fermentation process can be further used by photo-fermentative bacteria to produce hydrogen [12, 25]. However, the effluent of a dark fermentation must be treated before being subjected to photo fermentation to meet metabolic conditions for effective photo fermentation. Treatments, including dilution, nutrient addition, pH adjustment, and centrifugation all require energy-intensive inputs and are costly. In contrast, the bioaugmentation of PNSB into dark-fermentation process does not require these treatment processes because dark- and photo-fermentative bacteria are cocultured in the same reactor. VFAs produced from dark fermentation are immediately converted to hydrogen by photo-fermentative bacteria. This can also prevent accumulation of VFAs in the medium [26]. Thus, the fermentation time of bioaugmentation approach can be shortened which leads to an improvement in hydrogen productivity. Acetate and butyrate, the soluble metabolites commonly found in dark fermentation, can be easily converted into acetyl units, without formation of pyruvate, during the metabolism of PNSB. The acetyl units are mainly converted to polyhydroxyalkanoates (PHAs) via 3 ketothiolase, acetoacetyl-CoA reductase, and PHA synthase resulting in an accumulation of PHAs in PNSB rather than hydrogen production [27, 28]. Among the VFAs produced by dark fermentation, lactate is found to be a more suitable substrate for hydrogen production by PNSB than acetate and butyrate since lactate can be directly converted to pyruvate via lactate dehydrogenase. Pyruvate can be further catalyzed for ATP production in the Krebs cycle in which ATP is necessary for hydrogen production process [28, 29]. Therefore, in this study, a bioaugmentation of lactic acid-producing bacteria (LAB), i.e., *Lactobacillus delbrueckii* ssp. *bulgaricus* TISTR 895 into photo-fermentation process from glucose by PNSB, *Rhodobacter sphaeroides* KKU-PS5, was conducted in order to achieve maximum hydrogen production. The strain TISTR 895 is a homo-fermentative LAB. It produces lactate as its sole end-product [30, 31], while the strain, KKU PS5, is able to produce hydrogen from lactate [32].

The homo-fermentative LAB theoretically produces two moles of lactic acid from one mole of glucose [Eq. (2)] in which glucose is catalyzed to pyruvate through glycolysis and then pyruvate is reduced to lactic acid by lactate dehydrogenase of LAB [33]. Thereafter, PNSB consumes lactate as an electron donor (Eq. 3). Electrons from lactate are transferred to oxidized form of ferredoxin [Fd_(ox)_] through a series of membrane-bound electron transport carrier molecules [8], and then Fd_(red)_ molecules are used to reduce protons to produce hydrogen by nitrogenase as shown in Eq. (4). Therefore, the overall maximum theoretical HY is as high as 12 mol H_2_/mol hexose as shown in Eqs. (2) and (3) [34].

2 $$C_{6} H_{{12}} O_{6} \xrightarrow[{Lactic{\text{ }}acid-producing{\text{ }}bacteria}]{}2C_{3} H_{6} O_{3}$$

3 $$2C_{3} H_{6} O_{3} + 6H_{2} O\xrightarrow[{Purple{\text{ }}non\text{-}sulfur{\text{ }}photosynthetic{\text{ }}bacteria}]{}12H_{2} + 6CO_{2}$$

4 $$(C_{3} H_{6} O_{3} )\xrightarrow[{ATP \uparrow }]{}Fd_{{(red)}} \xrightarrow[{ATP \uparrow }]{}Nitrogenase \to H_{2}$$

A cultivation of two different organisms in the same bioreactor with proper conditions for both types of bacteria is difficult. This is because they are different in their metabolic types, optimal conditions, and nutritional requirements. The ratio of the number of dark- and photo-fermentative bacteria is an important factor for obtaining an appropriate match in growth and substrate utilization, which directly affects the rate of hydrogen production and the HY [35]. Initial cell concentration has a dramatic effect on the HPR. Too low a cell concentration causes a long lag time and slow fermentation, while too high a cell concentration causes a low HY [36]. The initial pH affects nitrogenase activity, the proton motive force of PNSB, and cell growth. These functions are responsible for hydrogen production [37]. Light intensity is another important factor since light is the sole energy source for ATP synthesis via photophosphorylation by PNSB during photo-hydrogen production under anaerobic conditions [38]. Molybdenum (Mo) is an important cofactor for the synthesis of nitrogenase [39, 40]. In light of the aforementioned information, there is a need to optimize these key factors to obtain suitable conditions for effective hydrogen production.

Hydrogen production from waste not only reduces pollution, but also reclaims renewable energy. However, the bottlenecks of hydrogen production from wastes are its low production rate and HY [41]. These problems may be solved by selecting and using effective organisms, an appropriate bacterial ratio, and cell concentration as well as suitable environmental conditions [41]. Therefore, the use of the optimal conditions in a bioaugmentation system well-characterized microorganisms using glucose as a model substrate can pave the way toward the development of an efficient hydrogen production process from sugar-containing wastes such as dairy industry residues, effluents of food processing plants, and sugar-refining (molasses) residues [5].

In this study, the important factors for bioaugmentation of PNSB, KKU-PS5, with LAB, TISTR 895, to produce hydrogen were optimized. The optimal LAB/PNSB bacterial ratio and initial cell concentration were investigated using a full factorial design followed by response surface methodology (RSM) with central composite design (CCD) for optimization of initial pH, light intensity, and Mo concentration.

## Results and discussion

### Optimization of LAB/PNSB ratio and initial cell concentration for bio-hydrogen production

Cumulative hydrogen production, HPR, and HY at different LAB/PNSB bacterial ratios and initial cell concentrations are given in Table 1. The results show that hydrogen was not produced at a LAB/PNSB ratio of 1/1 (Condition A) at any initial cell concentration (Conditions A1–A5). This is because LABs grow very much faster than PNSBs. Hence, at a LAB/PNSB ratio of 1/1, PNSBs were unable to consume lactic acid rapidly enough to prevent its accumulation. Accumulation of lactic acid can reduce the pH low enough to adversely affect the growth of PNSBs and its consumption of VFAs, as well as inhibiting nitrogenase [15]. When the LAB/PNSB ratio was 1/2 (Condition B), hydrogen production occurred at the initial cell concentration of 0.05 (Condition B1) and 0.10 g/L (Condition B2). However, when the initial cell concentration was further increased to 0.15 (Condition B3), 0.20 (Condition B4), and 0.25 g/L (Condition B5), hydrogen was not produced. At a low initial cell concentration of 0.05 (Condition B1) and 0.10 g/L (Condition B2), lactic acid in the fermentation broth was at concentrations of 0.4 and 0.6 g/L, respectively, while at a higher initial cell concentration of 0.15–0.25 g/L (Conditions B3–B5), lactic acid concentration in the fermentation broth was high (1.7–2.3 g/L) (Additional file 1). These results implied that when the initial cell concentrations were increased, the consumption rate of lactic acid by PNSB was lower than the rate of lactic acid production by LAB, which led to lactic acid accumulation, resulting in a decrease in hydrogen production.

**Table 1:** Cumulative hydrogen production, hydrogen production rate (HPR), and hydrogen yield (HY) at different lactic acid-producing bacteria/purple non-sulfur photosynthetic bacteria (LAB/PNSB) ratios and initial cell concentration

Condition	LAB/PNSB ratio (w/w)	Initial cell conc. (g/L)	LAB conc. (g/L)	PNSB conc. (g/L)	Cumulative H_2_ production (ml H_2_/L)	HPR (ml H_2_/L h)	HY (mol H_2_/mol glucose)
A							
A1	1:1	0.05	0.025	0.025	0	0	0
A2		0.10	0.050	0.050	0	0	0
A3		0.15	0.075	0.075	0	0	0
A4		0.20	0.100	0.100	0	0	0
A5		0.25	0.125	0.125	0	0	0
B							
B1	1:2	0.05	0.017	0.033	1355 ± 113	2.1 ± 0.2	3.01 ± 0.25
B2		0.10	0.033	0.067	721 ± 65	1.3 ± 0.1	1.58 ± 0.11
B3		0.15	0.050	0.100	0	0	0
B4		0.20	0.067	0.133	0	0	0
B5		0.25	0.083	0.167	0	0	0
C							
C1	1:7	0.05	0.066	0.044	1662 ± 41	3.1 ± 0.1	4.84 ± 0.11
C2		0.10	0.013	0.088	1460 ± 107	2.9 ± 0.2	4.00 ± 0.34
C3		0.15	0.019	0.131	1336 ± 56	2.7 ± 0.2	3.65 ± 0.25
C4		0.20	0.025	0.175	1403 ± 153	2.6 ± 0.3	3.39 ± 0.41
C5		0.25	0.031	0.219	1359 ± 129	2.6 ± 0.2	3.12 ± 0.20
D							
D1	1:12	0.05	0.004	0.046	1613 ± 69	3.1 ± 0.1	4.44 ± 0.19
D2		0.10	0.008	0.092	1590 ± 38	3.0 ± 0.1	4.47 ± 0.11
D3		0.15	0.012	0.138	1833 ± 72	3.6 ± 0.3	5.93 ± 0.23
D4		0.20	0.015	0.185	1587 ± 86	3.6 ± 0.2	5.84 ± 0.30
D5		0.25	0.019	0.231	1573 ± 96	3.6 ± 0.2	5.82 ± 0.32

At a LAB/PNSB ratio of 1/7 (Condition C), hydrogen was produced at every initial cell concentration (Conditions C1–C5). However, HPR and HY decreased with an increase in initial cell concentration from 0.05 (Condition C1) to 0.25 g/L (Condition C5). Cumulative hydrogen production, HPR, and HY were maximized at an initial cell concentration of 0.05 g/L (Condition C1). We found that at an initial cell concentration of 0.05 g/L (Condition C1), lactic acid concentration in fermentation broth was very low at 0.03 g/L, while at a high initial cell concentration of 0.10–0.25 g/L (Conditions C2–C5), lactic acid concentration was 10–14 times higher (Additional file 2). These results caused low hydrogen production due to the negative effect of high lactic acid concentration on hydrogen production. Even though the amount of PNSB increased with an increase in the amount of LABs at the same bacterial ratio, hydrogen production was decreased. Therefore, when two cultures were used to produce hydrogen, not only the optimal LAB/PNSB ratio, but also the initial cell concentration should be optimized in order to overcome lactic acid accumulation in the fermentation system.

**Fig. 1:**
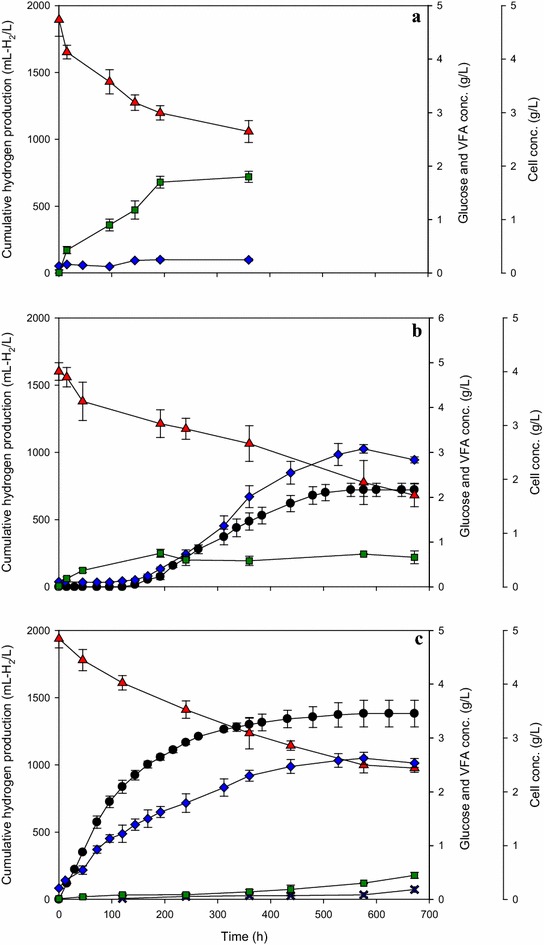
Variations of cumulative hydrogen production, concentration of cell, glucose, and volatile fatty acids over time under various lactic acid-producing bacteria/purple non-sulfur photosynthetic bacteria (LAB/PNSB) ratios and a fix LAB concentration of 0.03 g/L. LAB only (LAB concentration = 0.03 g/L) (**a**); LAB/PNSB ratio of 1/2 (LAB concentration = 0.033 g/L, PNSB concentration = 0.067 g/L) (**b**); LAB/PNSB ratio of 1/7 (LAB concentration = 0.031 g/L, PNSB concentration = 0.219 g/L) (**c**). Hydrogen (*black circle*), glucose (*red triangle*), cell (*blue diamond*), lactic acid (*green square*), formic acid (*blue cross*)

Based on our findings, we further investigated the effect of PNSB concentration on hydrogen production at a fixed initial LAB concentration of 0.03 g/L and LAB/PNSB ratios of 1/2 and 1/7. Figure 1 shows the time-course profiles of hydrogen production, cell, glucose, and metabolite (lactic acid, formic acid) concentrations of LAB fermentation at an initial LAB concentration of 0.03 g/L (Fig. 1a); PNSB augmented with LAB at a LAB/PNSB ratio of 1/2 with a LAB concentration of 0.033 g/L and a PNSB concentration of 0.067 g/L (Fig. 1b) (Condition B2); and PNSB augmented with LAB at a LAB/PNSB ratio of 1/7 with a LAB concentration of 0.031 g/L and a PNSB concentration of 0.219 g/L (Fig. 1c) (Condition C5). Approximately 1.8 g/L of lactic acid was produced by LAB (Fig. 1a) with 2.1 g/L of glucose utilized, or 86 % of the substrate was consumed for lactic acid formation by LAB (Fig. 1a). LAB concentration was increased from 0.03 to approximately 0.25 g/L. The results suggested that glucose was consumed by LAB to produce lactic acid and to maintain the cells without hydrogen production. Augmentation of LAB in the system at a LAB/PNSB ratio of 1/2 produced hydrogen at 721 ± 65 mL H_2_/L (Fig. 1b). Under this condition, lactic acid accumulated in the fermentation broth to about 0.66 ± 0.06 g/L, indicating that the amount of PNSB (0.067 g/L) added in the system was not high enough to balance the rate of lactic acid production and its consumption. When the concentration of PNSB was increased to 0.219 g/L at a LAB/PNSB ratio of 1/7 (Fig. 1c), hydrogen production increased to 1359 ± 129 mL H_2_/L, which was approximately two times higher than when the LAB/PNSB ratio was 1/2. The concentration of residual lactic acid in the fermentation broth was low. In addition, not only lactic acid was used as substrate to produce hydrogen by PNSB, but also glucose. Increasing the PNSB level at this LAB/PNSB ratio produced a higher lactic acid consumption rate, which in turn reduced lactic acid accumulation. Therefore, at a fixed LAB concentration, the cell concentration of PNSB could become a limiting factor for hydrogen production in a bioaugmentation system.

Maximum hydrogen production was obtained at a LAB/PNSB ratio of 1/12 (Condition D). Cumulative hydrogen production, HPR, and HY were increased as the initial cell concentration was increased from 0.05 to 0.15 g/L (Conditions D1–D3). However, hydrogen production decreased, while the HPR remained the same when the initial cell concentration was greater than 0.15 g/L (Conditions D4, D5) (Table 1). A maximum cumulative hydrogen production, HPR, and HY of 1833 ± 72 mL H_2_/L, 3.6 ± 0.3 mL H_2_/L h and 5.93 ± 0.23 mol H_2_/mol glucose, respectively, were obtained at an initial cell concentration of 0.15 g/L (Condition D3). These results indicate that at a LAB/PNSB ratio of 1/12, the amounts of LAB and PNSB were suitable for balancing the rate of lactic acid production by LAB and the rate of lactic acid consumption by PNSB without lactic acid accumulation. A decrease in hydrogen production at initial cell concentrations less than 0.15 g/L, at a LAB/PNSB ratio of 1/12, may be due to low cell concentrations. Furthermore, the reduction of hydrogen production seen at initial cell concentrations above 0.15 g/L may have been caused by consumption of available lactic acid and glucose for cell growth [15]. In addition, the shading effect caused by high cell concentrations could lead to a low hydrogen production [42].

The optimal dark and photo bacterial ratios are quite different from study to study. Liu et al. [15] reported that an optimal dark/photo bacterial ratio of *C. butyricum*/*Rhodopseudomonas faecalis* RLD-53 for maximal hydrogen production was 1/2. Argun et al. [43] reported that a dark/photo bacterial ratio (anaerobic sludge/mixture of *Rhodobacter* sp.) of 1/2 gave the lowest hydrogen production, while a dark/photo bacterial ratio of 1/7 was a suitable ratio. The differences in types of microorganisms, substrates used, and culture conditions may have contributed to these findings.

### Hydrogen production under the optimal LAB/PNSB ratio and initial cell concentration

**Fig. 2:**
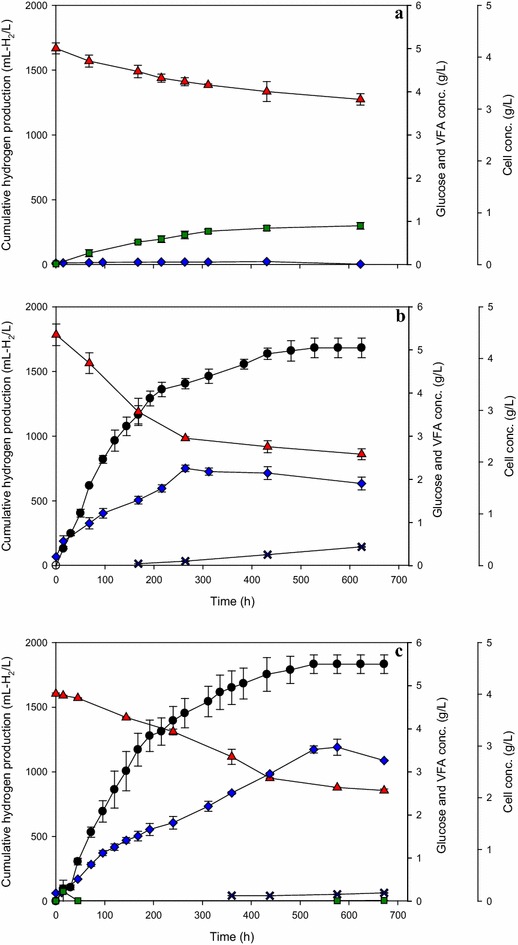
Variations of cumulative hydrogen production, concentrations of cell, glucose, and volatile fatty acid over time. Lactic acid-producing bacteria (LAB) only (LAB concentration = 0.019 g/L) (**a**); purple non-sulfur photosynthetic bacteria (PNSB) only (PNSB concentration = 0.131 g/L) (**b**); LAB/PNSB ratio of 1/12 at initial cell concentration of 0.15 g/L (LAB concentration = 0.019, PNSB concentration = 0.131 g/L) (**c**). Hydrogen (*black circle*), glucose (*red triangle*), cell (*blue diamond*), lactic acid (*green square*), formic acid (*blue cross*)

We conducted experiments to investigate the effects of LAB only, PNSB only, and PNSB augmented with LAB on cumulative hydrogen production, biomass, glucose, and VFA concentrations under the optimal LAB/PNSB ratio of 1/12 and an initial cell concentration of 0.15 g/L. The results demonstrated that LABs cultured alone consumed glucose for cell maintenance and produced lactic acid as their primary product without hydrogen production (Fig. 2a). PNSB cultured alone consumed glucose for cell growth and hydrogen production with the cumulative hydrogen production, HPR, and HY of 1682 ± 76 mL H_2_/L, 3.2 ± 0.1 mL H_2_/L h, and 4.38 ± 0.20 mol H_2_/mol glucose, respectively (Fig. 2b). Formic acid was found in the hydrogen fermentation by PNSB (Fig. 2b). Normally, under light illumination, PNSB will not produce formic acid. However, our results showed that formic acid was produced at approximately 0.4 g/L (Fig. 2b) in the PNSB only fermentation. This may have been caused by high cell concentrations (approximately 1.8 g/L) (Fig. 2b) which could make the medium opaque, restricting light from entering the serum bottle, resulting in a light-limited condition in the system. Therefore, the metabolism of KKU-PS5 to metabolize glucose might have adapted to dark or limited light conditions resulting in formic acid formation. Our results are similar to the findings of Eroglu et al. [44] who found that under limited illumination, hydrogen was not produced by *R. sphaeroides*, but formate was produced as an end-product.

In the experiment with PNSB augmented with LAB (Fig. 2c), the concentration of formic acid in the fermentation broth was less than the treatment with PNSB alone (Fig. 2b). The HY of 5.93 ± 0.23 mol H_2_/mol glucose obtained from the PNSB augmented with LAB treatment was higher than the PNSB-alone treatment, 4.38 ± 0.20 mol H_2_/mol glucose. The presence of formic acid is probably responsible for a low HY in the PNSB-alone treatment due to pyruvate conversion to formate rather than acetyl-CoA, resulting in conservation of less energy (ATP) and a lower concentration of (reduced) NAD(P)H which are important energy and electron carriers used for hydrogen production by photo-fermentative processes [29]. When PNSB was grown alone, cells grew to a stationary phase after 264 h with a cell concentration of approximately 1.8 g/L. A small amount of glucose was continuously consumed for hydrogen production in this period (264–624 h). In PNSB augmented with LAB system (Fig. 2c), the amount of bacterial cells was higher than that for LAB alone and PNSB alone. Cell growth continued after 264 h of fermentation with glucose utilization and hydrogen production. PNSB might be able to use lactic acid as a substrate for cell growth and hydrogen production during this period (264–624 h).

Our results suggested that glucose was used as the major substrate to produce hydrogen by PNSB and a bioaugmentation system. Glucose was consumed and reduced to approximately 2.6 g/L in both PNSB-alone and PNSB augmented with LAB systems. Considering an augmentation of LAB into PNSB system, lactate is likely an intermediate being transferred from LAB to PNSB for hydrogen production; therefore, a bioaugmentation system with a presence of potential lactate gave a slightly higher cumulative hydrogen production (1833 ± 72 mL H_2_/L), HY, and HPR (Fig. 2c) than the PNSB alone. These results indicate that the presence of LAB in the fermentation system is beneficial to hydrogen production in terms of serving lactate as another substrate for hydrogen production.

A low concentration of lactic acid detected in the fermentation broth indicated a suitable LAB/PNSB ratio to balance the rate of lactic acid production and its consumption (Fig. 2c). This result shows that hydrogen production by KKU-PS5 (PNSB) can be improved by the augmentation with TISTR 895 (LAB).

### Optimization of initial pH, light intensity, and Mo concentration on HPR by PNSB augmented with LAB

The effects of initial pH (*X*_1_), light intensity (*X*_2_), and Mo concentration (*X*_3_) on HPR by PNSB augmented with LAB were examined under the optimal LAB/PNSB ratio of 1/12 and initial cell concentration of 0.15 g/L. The predicted values of the response (HPR) in Table 2 were calculated using a quadratic equation (Eq. 5), which included the main effects, interaction effects, and the squared effects.

**Table 2:** Central composite experimental design (CCD) matrix defining initial pH (*X*_1_), light intensity (*X*_2_), Mo concentration (*X*_3_), and results on hydrogen production rate (HPR)

Run	Parameters						HPR (ml H_2_/L h)	
	Initial pH (*X*_1_)		Light intensity (*X*_2_)		Mo concentration (*X*_3_)		Observed	Predicted
	Code	Actual	Code	Actual (klux)	Code	Actual (mg/L)		
1	0.00	8.00	0.00	8.00	0.00	0.30	8.9 ± 0.4	8.8
2	−1.00	7.00	1.00	10.00	1.00	0.50	7.4 ± 0.6	7.3
3	0.00	8.00	0.00	8.00	0.00	0.30	8.8 ± 0.2	8.8
4	−1.68	6.32	0.00	8.00	0.00	0.30	5.6 ± 0.5	5.7
5	0.00	8.00	1.68	11.36	0.00	0.30	7.5 ± 0.1	7.7
6	0.00	8.00	−1.68	4.64	0.00	0.30	5.8 ± 0.4	6.5
7	0.00	8.00	0.00	8.00	0.00	0.30	8.8 ± 0.3	8.8
8	1.00	9.00	−1.00	6.00	−1.00	0.10	6.2 ± 0.2	5.7
9	0.00	8.00	0.00	8.00	0.00	0.30	9.0 ± 0.6	8.8
10	0.00	8.00	0.00	8.00	0.00	0.30	9.1 ± 0.4	8.8
11	1.00	9.00	−1.00	6.00	0.20	0.50	6.6 ± 0.1	6.0
12	1.00	9.00	1.00	10.00	−1.00	0.10	6.8 ± 0.4	6.5
13	0.00	8.00	0.00	8.00	0.00	0.30	8.5 ± 0.4	8.8
14	−1.00	7.00	1.00	10.00	−1.00	0.10	7.3 ± 0.5	7.3
15	−1.00	7.00	−1.00	6.00	−1.00	0.10	6.7 ± 0.4	6.5
16	−1.00	7.00	−1.00	6.00	1.00	0.50	7.0 ± 0.2	6.7
17	1.00	9.00	1.00	10.00	1.00	0.50	7.0 ± 0.4	6.5
18	0.00	8.00	0.00	8.00	1.68	0.64	7.5 ± 0.4	8.0
19	1.68	9.68	0.00	8.00	0.00	0.30	3.5 ± 0.2	4.3
20	0.00	8.00	0.00	8.00	−1.68	−0.04	7.4 ± 0.0	7.8

5 $$Y_{{{\text{HPR}}}} = 8.82{\mkern 1mu} {\mkern 1mu} - {\mkern 1mu} {\mkern 1mu} 0.40X_{1} + 0.35X_{2} + 0.078X_{3} {\mkern 1mu} {\mkern 1mu} - {\mkern 1mu} {\mkern 1mu} 0.012X_{1} X_{2} + 0.013{\mkern 1mu} X_{1} X_{3} {\mkern 1mu} {\mkern 1mu} - {\mkern 1mu} {\mkern 1mu} 0.062{\mkern 1mu} X_{2} X_{3} {\mkern 1mu} {\mkern 1mu} - {\mkern 1mu} {\mkern 1mu} 1.35{\mkern 1mu} X_{1}^{2} {\mkern 1mu} {\mkern 1mu} - {\mkern 1mu} {\mkern 1mu} 0.61{\mkern 1mu} X_{2}^{2} {\mkern 1mu} {\mkern 1mu} - {\mkern 1mu} {\mkern 1mu} 0.32{\mkern 1mu} X_{3}^{2}$$

The coefficient of determination, *R*^2^, was 0.9178, suggesting the model could explain 91.78 % of the variation in the response. This indicated a good fit to the experimental data. The *p* value (*p* = 0.0003) obtained from the regression analysis of variance (ANOVA) was less than 0.05 indicating the significance of the model (Table 3). The significance of each coefficient was determined using probability values. Linear terms of initial pH (*X*_1_) and light intensity (*X*_2_) showed significant individual effects on HPR (*p* ≤ 0.05), whereas Mo supplementation had no significant effect on HPR (*p* = 0.6118). All of interaction terms (*X*_1_*X*_2_, *X*_1_*X*_3_, *X*_2_*X*_3_) had *p* values higher than 0.05, indicating no significant interaction effects between variables on HPR. The quadratic terms of these three factors (*X*_1_^2^, *X*_2_^2^, *X*_3_^2^) were significant (*p* ≤ 0.05). The optimal conditions for hydrogen production in bioaugmentation system that maximized HPR were obtained from the analysis of Eq. (5). The predicted maximum response value for HPR was 8.77 mL H_2_/L h at an initial pH of 7.92, light intensity of 8.37 klux, and Mo concentration of 0.44 mg/L.

**Table 3:** ANOVA of the fitting model for hydrogen production rate (HPR)

Source	Sum of squares	*df*	Mean of square	*F*-value	(Probability) probe >*F*
Model	33.80	9	3.76	12.33	0.0003
X_1_	2.16	1	2.16	7.09	0.0238
X_2_	1.66	1	1.66	5.45	0.0418
X_3_	0.084	1	0.084	0.27	0.6118
X_1_X_2_	0.00125	1	0.00125	0.0041	0.9502
X_1_X_3_	0.00125	1	0.00125	0.0041	0.9502
X_2_X_3_	0.031	1	0.031	0.10	0.7553
X_1_^2^	26.28	1	26.28	86.28	<0.0001
X_2_^2^	5.32	1	5.32	17.48	0.0019
X_3_^2^	1.52	1	1.52	5.00	0.0494
Residual	3.05	10	0.30		
Lack of fit	2.83	5	0.57	13.16	0.0067
Cor total	36.85	19			
Coefficient of determination (*R* ^2^) = 0.9178 Adjusted determination coefficient (adj *R* ^2^) = 0.8434					

**Fig. 3:**
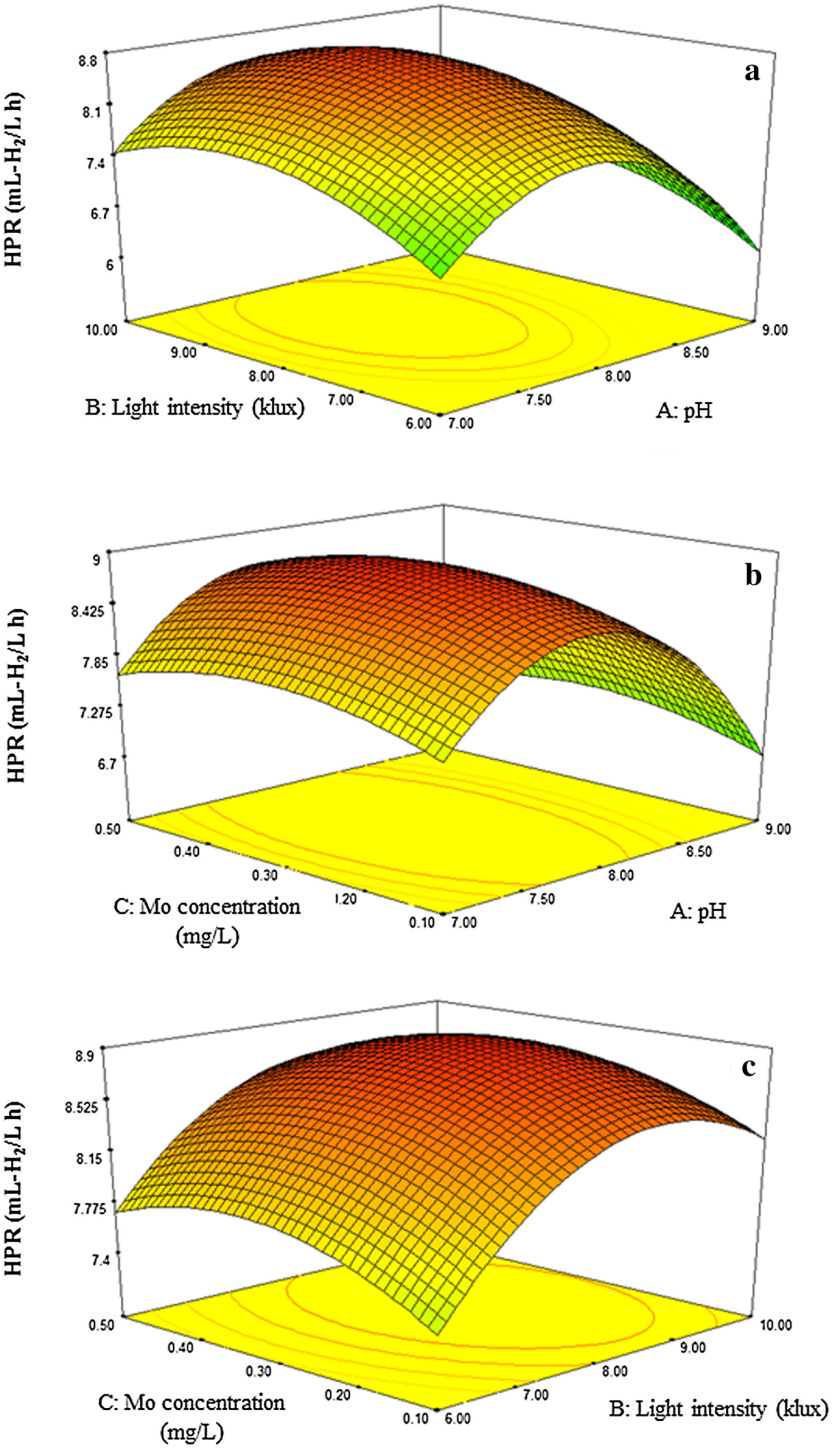
Response surface plots showing the effects of initial pH, light intensity, and Mo concentration on hydrogen production rate (HPR). The interactive effect of light intensity and pH at a fixed the amount of Mo concentration of 0.44 mg/L (**a**); the interactive effect of Mo concentration and pH at a fixed light intensity of 8.37 klux (**b**); the interactive effect of Mo concentration and light intensity at a fixed pH of 7.92 (**c**)

Response surface plots in three dimensions were developed based on Eq. (5) with one variable being kept constant at its optimal level, and varying the other two parameters over the experimental range (Fig. 3a–c). The highest points in Fig. 3 indicate the optimal conditions for maximal HPR. The HPR increased with the increasing light intensity from 6.00 to 8.37 klux and decreased at light intensities over 8.37 klux (Fig. 3a, c). Light provides ATP and reductive power to the photosynthetic system of photo-fermentative bacteria needed for the hydrogen production process [45]. However, excess light causes a saturation effect, in which ATP and Fd_(red)_ were excessive for the available nitrogenase [2]. In addition, excess protons generated under high light intensities were captured by photo-fermentative bacteria and dissipated as heat energy, damaging their photosynthetic apparatus [46]. Consequently, a low HPR was obtained at high light intensity. Our previous research found that the optimal light intensity for the strain KKU-PS5 was 6 klux [32]. The higher optimal light intensity, from 6 to 8.37 klux, found in this study may be due to a shading effect of LAB and PNSB in the fermentation broth. Hence, higher light intensity is needed for hydrogen fermentation by bioaugmentation system than that by PNSB alone.

Figure 3a and b indicates that initial pH had a great influence on HPR. When the initial pH increased from 7.00 to 7.92, the HPR increased and then markedly decreased when initial pH was further increased over 7.92. This revealed that the suitable initial pH for hydrogen production from glucose by PNSB augmented with LAB was 7.92. The initial pH had a great influence on the lag period of hydrogen production. Kim et al. [47] found that if the fermentation process was started at an optimal initial pH, a short lag phase would be achieved. In this study, during the fermentation process, the pH was decreased due to the production of lactic and formic acids by both types of bacteria. The final pH was in the range of 6.7–7.0, when the fermentation was conducted at an initial pH of 8.0 and 9.0. The final pH was decreased to about 6.3–6.6 when the initial pH was 7.0 (Additional file 3). A pH in the range of 6.3–6.6 was found to fall in an optimal pH range of LAB metabolism for lactic acid production [48, 49] leading to a high lactic acid production rate, causing a pH drop. This adversely affected photo-fermentative bacterial growth and hydrogen production. When the fermentation process was conducted at a higher initial pH than 7.92, hydrogen production was decreased due to low proton motive force. Consequently, ATP in the cell was lowered inhibiting bacterial growth [50]. In addition, the efficiency of nitrogenase decreases at alkaline pHs [51] resulting in less hydrogen production. Thus, the pH of the medium during the fermentation process is considered an important factor for enhancing hydrogen production. When pH is appropriately controlled, effective hydrogen production can be obtained.

HPR slightly increased with an increase in the amount of Mo supplementation from 0.1 to 0.44 mg/L (Fig. 3b, c). Availability of Mo was found to be important since it is a required cofactor of Mo-nitrogenase [52, 53] responsible for hydrogen production by photo-fermentative bacteria. Mo-nitrogenases consist of two metalloprotein components, i.e., dinitrogenase or molybdenum-iron (Mo-Fe) protein (encoded by *nif*D and *nif*K) and the dinitrogenase reductase or Fe protein (encoded by *nifH*) [54]. Electron transfer from the Fe protein to the Mo-Fe protein is facilitated by the hydrolysis of ATP molecules involved in hydrogen production mechanisms of Mo-nitrogenases [55]. Mo is a cofactor that is incorporated in Mo-Fe protein and is required for completing the function of Mo-nitrogenase for hydrogen production [55]. This is evidenced by the study of Kars et al. [56] who found that significant *nifK* gene expression in a medium supplement with Mo resulted in enhanced hydrogen production. However, differences in the optimal Mo concentration were reported. This may have been due to differences in microbial types, culture conditions, and experimental range. An optimal Mo concentration of 0.8 mg/L was favorable for hydrogen production by *Rhodospeudomonas palustris* KU003 [57], while Mo concentrations of 0.02 and 1.58 mg/L were optimal for *Rhodobacter capsulatus* and *R. sphaeroides* O.U.001, respectively [40, 56].

### Confirmation experiment

The model (Eq. 5) was used to predict optimal initial pH, light intensity, and Mo concentration in order to obtain the maximum HPR. The predicted optimal conditions were an initial pH of 7.92, light intensity of 8.37 klux, and a Mo concentration of 0.44 mg/L, at which the maximum predicted HPR was 8.8 mL H_2_/L h. Three replicate batch fermentations with five experiments each were conducted under the optimal, low (run 15), high (run 17), and central conditions (runs 1, 3, 7, 9, 10, 13) (Table 4) to confirm the validity of the model obtained. A cumulative hydrogen production, HPR, and HY of 3396 ± 66 mL H_2_/L, 9.1 ± 0.2 mL H_2_/L h, and 9.65 ± 0.23 mol H_2_/mol glucose, respectively, were obtained under these conditions. The observed HPR was 9.1 ± 0.2 mL H_2_/L h which was within 3.3 % of the predicted value (8.8 mL H_2_/L h). This indicated high model validity in the CCD experiment.

**Table 4:** Confirmation hydrogen production experiments

Run	Condition	X_1_ initial pH	X_2_ light intensity (klux)	X_3_ Mo concentration (mg/L)	HPR (ml H_2_/L h)	HY (mol H_2_/mol glucose)	Cumulative H_2_ production (ml H_2_/L)
1	Optimum	7.92	8.37	0.44	9.1 ± 0.2	9.65 ± 0.23	3396 ± 66
2	Lower	7.00	6.00	0.10	6.3 ± 0.1	8.14 ± 0.12	2726 ± 39
3	Upper	9.00	10.00	0.50	6.9 ± 0.1	8.20 ± 0.20	2585 ± 53
4	Central	8.00	8.00	0.30	8.7 ± 0.1	9.46 ± 0.19	3253 ± 20

*HPR* hydrogen production rate, *HY* hydrogen yield

### Comparison of hydrogen production to the literature search

The HPR and HY in this study were compared with previous reports that used dark- and photo-fermentative bacteria for hydrogen production. The large variations in the HPR in the literature were found in the range of 0.04–100 ml H_2_/L h (Table 5). The HPR of 9.1 ± 0.2 mL H_2_/L h obtained in this study was markedly higher than that obtained by Kuo et al. [17] and Qin et al. [22]. However, our HPR was lower than that reported by Sivagurunathan et al. [16], Goud et al. [16], and Marone et al. [18]. This was due to the fact that these researchers used dark-fermentative bacteria capable of producing hydrogen to enhance HPR. The microorganisms used in the studies included *Enterobacter cloacae* DSM 16657, *Escherichia coli* XL1-BLUE, *Bacillus subtilis*, *Pseudomonas stutzeri*, *Lysinibacillus fusiformis*, *Buttiauxella* sp. 4, *Rahnella* sp. 10, and *Raoultella* sp. 47. Therefore, the discrepancy in HPR is due to the type of bacteria, the cultivation conditions, and substrate used.

**Table 5:** Hydrogen production rate (HPR) and hydrogen yield (HY) by different bioaugmentation experiments

Augmented microorganisms	Native microorganisms	Substrate	HPR	HY	Ref.
*Enterobacter cloacae* (DSM 16657) *Escherichia Coli* XL1-BLUE Mix consortium Non-augmentation	Anaerobic-enriched mixed cultures (EMC) from compost of food waste	Beverage wastewater (10 g glucose equivalent/L)	93.8 ml H_2_/L h 51.0 ml H_2_/L h 70.1 ml H_2_/L h 75.6 ml H_2_/L h	0.84^a^ mol H_2_/mol glucose 0.63^a^ mol H_2_/mol glucose 0.70^a^ mol H_2_/mol glucose 1.13^a^ mol H_2_/mol glucose	[23]
*Bacillus subtilis*, *Pseudomonas stutzeri*, *Lysinibacillus fusiformis*, non-augmentation	Anaerobic consortium collected from a full scale anaerobic reactor treating composite wastewater	Real-field food wastewater (50 g COD/L)	52.1^a^ ml H_2_/L h 19.8^a^ ml H_2_/L h 24.8^a^ ml H_2_/L h 5.0^a^ ml H_2_/L h	3.8^a^ mol H_2_/kg COD 1.4^a^ mol H_2_/kg COD 1.9^a^ mol H_2_/kg COD 0.4^a^ mol H_2_/kg COD	[16]
Clostridium sp. TCW1 semicontinuous culture fed three times daily Before augmentation	Enriched cellulolytic bacteria (*Bacillus thermoamylovorans* 9-4AIA and *Clostridium thermocellum* DSM 1313)	Vegetable-based kitchen waste and Napier grass (20 g COD/m^3^ day)	4.6^a^ ml H_2_/L h 2.1^a^ ml H_2_/L h	14 mol H_2_/kg COD_in_ 6 mol H_2_/kg COD_in_	[17]
*Buttiauxella* sp. 4, *Rahnella* sp. 10, *Raoultella* sp. 47, Mix consortium Non-augmentation	Indigenous microbial communities in vegetable waste	Vegetable waste	60^a^ ml H_2_/L h 64^a^ ml H_2_/L h 60^a^ ml H_2_/L h 100^a^ ml H_2_/L h 24^a^ ml H_2_/L h	71.3 ml H_2_/g VS 47.5 ml H_2_/g VS 69.7 ml H_2_/g VS 85.7 ml H_2_/g VS 21.9 ml H_2_/g VS	[18]
*Ethanoigenens harbinense* B49 Non-augmentation (strain X9 only)	*Clostridium acetobutylicum* X9	Dark fermentation of microcrystal-line cellulose (10 g/L)	–	1810 ml H_2_/L 755 ml H_2_/L	[20]
Selectively enriched kanamycin-resistant mixed consortia	Native anaerobic mixed microflora in chemical wastewater	Chemical wastewater treatment	0.02 mol H_2_/kg COD h	–	[21]
Non-augmentation			0.012 mol H_2_/kg COD h		
Ethanoligenens sp. B49 Non-augmentation	Activated sludge	– (OLR 12 kg/m^3^.d)	0.07^a^ ml H_2_/L h 0.04^a^ ml H_2_/L h	–	[22]
*Lactobacillus delbrueckii ssp. balgaricus* TISTR 895 Non-augmentation (strain KKU-PS5 only) Strain TISTR 895 under optimal condition	*Rhodobacter sphaeroides* KKU-PS5	Glucose (5 g/L)	3.6 ± 0.3 ml H_2_/L h 3.2 ± 0.1 ml H_2_/L h 9.1 ± 0.2 ml H_2_/L h	5.93 ± 0.23 mol H_2_/mol glucose 4.38 ± 0.20 mol H_2_/mol glucose 9.65 ± 0.23 mol H_2_/mol glucose	This study

^a^The values obtained from calculation according to the data given in the article

The HY obtained in this study (9.65 ± 0.23 mol H_2_/mol glucose) is higher than that reported by Sivagurunathan et al. [23] but could not be compared with other study due the difference in the unit. However, the HY achieved in this study was 80 % of the theoretical HY which is the highest value that has previously been reported. Keasling et al. [58] suggested that a HY of at least 8 mol H_2_/mol hexose is sufficient for economic applications. The high HY obtained could have been due to the use of glucose as the substrate, which is a less complex carbon source. Another reason was due to the fact that glucose was fermented to lactic acid by augmented LAB. As we know that lactic acid is a favored substrate for hydrogen production by PNSB, the HY in the LAB-augmented PNSB system was relatively high in our study. Our results indicated that by optimizing the key factors affecting hydrogen production of these two bacteria, the maximum hydrogen production could be achieved.

## Conclusions

The ratio of LAB/PNSB and the initial cell concentrations showed interactive effects on the rate of lactic acid production and its consumption. A suitable LAB/PNSB ratio and initial cell concentration could balance lactic acid production rate and its consumption to avoid lactic acid accumulation in the fermentation system. A suitable LAB/PNSB ratio and initial cell concentration were found to be 1/12 (w/w) and 0.15 g/L, respectively. The optimal initial pH, light intensity, and Mo concentration obtained from RSM with CCD were 7.92, 8.37 klux, and 0.44 mg/L, respectively. Under these optimal conditions, cumulative hydrogen production of 3396 ± 66 mL H_2_/L and HPR of 9.1 ± 0.2 mL H_2_/L h were obtained. The observed HPR under the optimal conditions (9.1 ± 0.2 mL H_2_/L h) was only 3.33 % different from the predicted HPR value (8.8 mL H_2_/L h). PNSB augmented with LAB produced hydrogen from glucose with a relatively high HY, 9.65 ± 0.23 mol H_2_/mol glucose, i.e., 80 % of the theoretical yield. The augmentation of LAB in the PNSB fermentation system is beneficial to hydrogen production in terms of serving lactate as another substrate for hydrogen production. By using appropriate environmental conditions for a cultivation of dark- and photo-fermentative bacteria, an improvement in hydrogen production can be achieved.

## Methods

### Media

The de Man, Rogosa, and Sharpe medium (MRS medium) for culturing lactic acid-producing bacteria was purchased from MERCK, Germany. The pH of the medium was adjusted to 6.8 using 2 N NaOH or 2 N HCl.

The growth medium for culturing PNSB was modified from RCVB medium [59]. The growth medium contained (all in g/L): lactic acid 2, sodium glutamate 0.68, yeast extract 1, KH_2_PO_4_ 0.4, MgSO_4_∙7H_2_O 0.4, NaCl 0.4, CaCl_2_ 0.05, FeSO_4_ 0.0005 in the form of an Fe-EDTA complex and trace elements 1 mL. The trace elements for the growth medium consisted of (all in mg/L): ZnCl_2_∙7H_2_O 100, MnCl_2_∙4H_2_O 30, H_3_BO_3_ 300, CoCl_2_∙6H_2_O 200, CuCl_2_∙2H_2_O 10, NiCl_2_∙6H_2_O 20, and Na_2_MoO_4_ 30 [58]. The pH of the growth medium was adjusted to 7.0 using NaOH (in pellet form).

The hydrogen production medium consisted of (all in g/L): glucose 5, sodium glutamate 0.68, K_2_HPO_4_ 2.8, KH_2_PO_4_ 3.9, MgSO_4_∙7H_2_O 0.2, CaCl_2_ 0.075, Na_2_MoO_4_ 0.02, and FeSO_4_ in the form of an Fe-EDTA complex 0.002 and trace elements (1 mL). The trace elements for the hydrogen production medium consisted of (all in mg/L): ZnCl_2_∙7H_2_O 100, MnCl_2_∙4H_2_O 30, H_3_BO_3_ 300, CoCl_2_∙6H_2_O 200, CuCl_2_∙2H_2_O 10, NiCl_2_∙6H_2_O 20 and Na_2_MoO_4_ 30.

### Bacterial strains and culture conditions

LAB, *L. delbrueckii* subsp. *bulgaricus* TISTR 895, was purchased from the Thailand Institute of Scientific and Technological Research (TISTR), Thailand. It was cultured in MRS medium for 12–14 h under static conditions at 35 °C in an incubator before being used as seed inoculum.

PNSB, *R. spaeroides* KKU-PS5, was previously isolated from an upflow anaerobic sludge blanket (UASB) bioreactor used to produce methane from a hydrogenogenic effluent [32]. Single colonies of the KKU-PS5 strain were grown in growth medium for 2 days, under 4 klux of light illumination using light emitting diode (LED) lamps, on an incubating shaker at 150 rpm. Then, a subculture was made in fresh growth medium and cultivated under the same conditions for 24 h, before being used as seed inoculum.

### Optimization of LAB/PNSB ratio and initial cell concentration for bio-hydrogen production

A full factorial design was used to obtain the optimal LAB/PNSB ratios and initial cell concentration for hydrogen production by bioaugmentation system. Dark and light fermentative bacteria were mixed at different ratios, 1:1, 1:2, 1:7, and 1:12 (w/w), for use as seed inocula. The inocula at each LAB/PNSB ratio were added into serum bottles containing hydrogen production medium at different initial cell concentrations (0.05, 0.10, 0.15, 0.20 and 0.25 g/L). The serum bottles were incubated at 30 °C, at 150 rpm on an incubating shaker under 4 klux of light illumination using LED lamps. Biogas samples were taken every 15 h during the first 2 days, then every 24 h until 16 days, and finally every 48 h thereafter until the end of fermentation. Fermentation broth was sampled at these intervals to determine the glucose, VFAs, and cell concentrations.

### Optimization of key factors affecting hydrogen production by bioaugmentation system

RSM with CCD was used to optimize the key factors affecting hydrogen production from glucose by bioaugmentation system. Twenty experimental runs with three replicates (Table 2) were generated. The key factors were initial pH (unit) (*X*_1_), light intensity (klux) (*X*_2_), and Mo concentration (mg/L) (*X*_3_). The response was HPR. Design-Expert software (Demo version 7.0, Stat-Ease, Inc., Minneapolis, MN, USA) was used to analyze the regression and graphically depict the experimental data. The quality fit of the model was determined by correlative coefficient value, *R*^2^, and its statistical significance was checked using the *F* test.

### Bio-hydrogen production

All batch hydrogen fermentations were conducted in 120-mL serum bottles with 70-mL working volumes. The serum bottles were flushed with argon gas for 5 min to create an anaerobic condition before being closed with rubber stoppers, capped with aluminum caps, and sterilized in an autoclave at 110 °C for 28 min. After inoculation, the serum bottles were placed on an incubating shaker running at 150 rpm under continuously lighted conditions using LED lamps. The volume of biogas in the head space of the serum bottles was measured using wetted glass syringes [60]. The biogas composition was analyzed using gas chromatography (GC). All experiments were done in triplicate.

### Analytic methods

Cell concentration was measured using a spectrophotometer (UVmini-1240, Shimadzu, Japan) at 620 nm for the strain TISTR 895 (1 unit of absorbance was equal to 0.4358 g dry cell/L) and 660 nm for the strain KKU-PS5 (1 unit of absorbance was equal to 0.3964 g dry cell/L). Cell concentration of the bioaugmentation system was measured at 660 nm (1 unit of absorbance was equal to 0.4057 g dry cell/L). The method to determine dry cell weight (g/L) was described by Laocharoen and Reungsang [32]. The pH was measured using a pH meter (Model pH500, Clean USA).

Hydrogen gas production was determined from measurement of gas composition and content of biogas in the head space of serum bottles. A mass balance was used to calculate the total volume of hydrogen [61]. Biogas compositions were determined using a GC (Shimadzu, GC-2014; Japan) equipped with a thermal conductivity detector (TCD) and a 0.2 m × 3 mm-diameter stainless column packed with Shin carbon (50/80 mesh). The temperatures of the injector port, column oven, and detector were 130, 120, and 140 °C, respectively. Helium was used as the carrier gas at a flow rate of 25 mL/min.

The concentrations of VFAs were determined using a high-performance liquid chromatograph (HPLC) (Shimadzu, DGU-20; Japan) equipped with a refractive index detector (RID) using an Aminex HPX-87H column. The HPLC conditions followed the method of Laocharoen and Reungsang [32].

HY (mol H_2_/mol substrate) was calculated as the total mol of hydrogen (mol H_2_) divided by the mol of glucose consumed (mol glucose). The HPR (mL H_2_/L h) was calculated from cumulative hydrogen production (mL H_2_/L) divided by fermentation time (h).

## Additional files

10.1186/s13068-015-0375-z Lactic acid concentration at lactic acid-producing bacteria/purple non-sulfur photosynthetic bacteria (LAB/PNSB) ratios of 1/2.
10.1186/s13068-015-0375-z Lactic acid concentration at lactic acid-producing bacteria/purple non-sulfur photosynthetic bacteria (LAB/PNSB) ratios of 1/7.
10.1186/s13068-015-0375-z Final pH at each run in central composite experimental design (CCD).

## Abbreviations

CCD: central composite design
COD: chemical oxygen demand
Fd_(od)_: oxidized ferredoxin
Fd_(red)_: reduced ferredoxin
GC: gas chromatography
HPLC: high-performance liquid chromatography
HPR: hydrogen production rate
HY: hydrogen yield
LAB: lactic acid-producing bacteria
*L. delbrueckii*: *Lactobacillus delbrueckii* ssp. *bulgaricus* TISTR 895
LED: light emitting diode
LH1: light-harvesting complex 1
LH2: light-harvesting complex 2
Mo: molybdenum
Mo-Fe: molybdenum-iron
NH^4+^-N: ammonia nitrogen
PHAs: polyhydroxyalkanoates
PHB: polyhydroxybutyrate
PNSB: purple non-sulfur photosynthetic bacteria
*R. sphaeroides* KKU-PS5: *Rhodobacter sphaeroides* KKU-PS5
RID: refractive index detector
RSM: response surface methodology
TCD: thermal conductivity detector
UASB: upflow anaerobic sludge blanket
VFAs: volatile fatty acids
