# Supplementary material for: Bioaugmentation of Lactobacillus delbrueckii ssp. bulgaricus TISTR 895 to enhance bio-hydrogen production of Rhodobacter sphaeroides KKU-PS5
Source: Biotechnol Biofuels. 2015 Nov 25;8:190. doi: 10.1186/s13068-015-0375-z (PMC4660636; doi:10.1186/s13068-015-0375-z)
Supplement: Supplementary file 1 — 10.1186/s13068-015-0375-z Lactic acid concentration at lactic acid-producing bacteria/purple non-sulfur photosynthetic bacteria (LAB/PNSB) ratios of 1/2. [file 13068_2015_375_MOESM1_ESM.pdf]

Condition		LAB/ PNSB ratio (w/w)	Initial Cell conc. (g/L)	LAB conc. (g/L)	PNSB conc. (g/L)	Lactic acid conc. (g/L)
B	B1	1:2	0.05	0.017	0.033	0.42±0.09
	B2		0.10	0.033	0.067	0.66±0.14
	B3		0.15	0.050	0.100	1.72±0.08
	B4		0.20	0.067	0.133	2.07±0.10
	B5		0.25	0.083	0.167	2.35±0.08
