# Supplementary material for: Bioaugmentation of Lactobacillus delbrueckii ssp. bulgaricus TISTR 895 to enhance bio-hydrogen production of Rhodobacter sphaeroides KKU-PS5
Source: Biotechnol Biofuels. 2015 Nov 25;8:190. doi: 10.1186/s13068-015-0375-z (PMC4660636; doi:10.1186/s13068-015-0375-z)
Supplement: Supplementary file 2 — 10.1186/s13068-015-0375-z Lactic acid concentration at lactic acid-producing bacteria/purple non-sulfur photosynthetic bacteria (LAB/PNSB) ratios of 1/7. [file 13068_2015_375_MOESM2_ESM.pdf]

Condition		LAB/ PNSB ratio (w/w)	Initial Cell conc. (g/L)	LAB conc. (g/L)	PNSB conc. (g/L)	Lactic acid conc. (g/L)
C	C1	1:7	0.05	0.066	0.044	0.03±0.001
	C2		0.10	0.013	0.088	0.31±0.02
	C3		0.15	0.019	0.131	0.43±0.03
	C4		0.20	0.025	0.175	0.44±0.01
	C5		0.25	0.031	0.219	0.45±0.05
