# Supplementary material for: Bioaugmentation of Lactobacillus delbrueckii ssp. bulgaricus TISTR 895 to enhance bio-hydrogen production of Rhodobacter sphaeroides KKU-PS5
Source: Biotechnol Biofuels. 2015 Nov 25;8:190. doi: 10.1186/s13068-015-0375-z (PMC4660636; doi:10.1186/s13068-015-0375-z)
Supplement: Supplementary file 3 — 10.1186/s13068-015-0375-z Final pH at each run in central composite experimental design (CCD). [file 13068_2015_375_MOESM3_ESM.pdf]

Run	Parameters						Final pH
	Initial pH (X_1)		Light Intensity (X_2)		Mo concentration (X_3)		
	Code	Actual	Code	Actual (klux)	Code	Actual (mg/L)	
1	0.00	8.00	0.00	8.00	0.00	0.30	6.86±0.01
2	-1.00	7.00	1.00	10.00	1.00	0.50	6.63±0.01
3	0.00	8.00	0.00	8.00	0.00	0.30	6.88±0.03
4	-1.68	6.32	0.00	8.00	0.00	0.30	5.25±0.04
5	0.00	8.00	1.68	11.36	0.00	0.30	6.86±0.05
6	0.00	8.00	-1.68	4.64	0.00	0.30	7.00±0.03
7	0.00	8.00	0.00	8.00	0.00	0.30	6.84±0.01
8	1.00	9.00	-1.00	6.00	-1.00	0.10	6.83±0.01
9	0.00	8.00	0.00	8.00	0.00	0.30	6.80±0.01
10	0.00	8.00	0.00	8.00	0.00	0.30	6.77±0.01
11	1.00	9.00	-1.00	6.00	0.20	0.50	6.78±0.02
12	1.00	9.00	1.00	10.00	-1.00	0.10	6.77±0.02
13	0.00	8.00	0.00	8.00	0.00	0.30	6.70±0.02
14	-1.00	7.00	1.00	10.00	-1.00	0.10	6.33±0.01
15	-1.00	7.00	-1.00	6.00	-1.00	0.10	6.53±0.01
16	-1.00	7.00	-1.00	6.00	1.00	0.50	6.60±0.05
17	1.00	9.00	1.00	10.00	1.00	0.50	6.83±0.01
18	0.00	8.00	0.00	8.00	1.68	0.64	6.78±0.04
19	1.68	9.68	0.00	8.00	0.00	0.30	6.93±0.03
20	0.00	8.00	0.00	8.00	-1.68	-0.04	6.83±0.01
